# Adult renal sarcoma: A rare case of recurrence 13 years after initial resection

**DOI:** 10.1002/ccr3.1911

**Published:** 2018-11-11

**Authors:** Mahmoud Alameddine, Ian Zheng, Ali Yusufali, Nicholas Mackrides, Gaetano Ciancio

**Affiliations:** ^1^ Department of Urology University of Miami Miller School of Medicine Miami Florida; ^2^ Department of Pathology University of Miami Miller School of Medicine Miami Florida

**Keywords:** liposarcoma, radical nephrectomy, renal sarcoma, soft tissue sarcoma

## Abstract

Renal sarcoma is a rare and aggressive malignancy without proper guidelines for treatment. Due to the aggressiveness of this disease and the potential for recurrence, we believe that extensive surgical resection with healthy margins may be the best option to treat this condition during both initial resection and resection of the recurrent lesion. Clinical follow‐up is also important to monitor for tumor recurrence.

## 1 INTRODUCTION

The majority of all renal cancers are attributed to renal carcinoma, with only 1% of all renal cancers arising from the connective tissue or renal sarcoma.^1^ Histological examination of a renal sarcoma shows mesenchymal differentiation which varies depending on subtype, and generally an absence of epithelial differentiation.^1^ Renal sarcoma is a very aggressive malignancy. The 5‐year overall survival rate for non‐metastatic renal sarcoma is 46% compared to 8% for metastatic disease.^1^ Due to the rarity of this malignancy, proper guidelines for treatment and course of action with renal sarcoma have not yet been established.

We present a novel case of a locally advanced renal sarcoma that recurred 13 years after its initial resection. The goal of our study is to improve understanding of the clinical and pathological characteristics of this disease, as well as pave the way for future surgical treatment options for patients presenting with this malignancy.

## 2 CASE REPORT

A 55‐year‐old patient initially presented with gross hematuria and left flank pain. His physical exam revealed a palpable left abdominal mass. Past medical history was consistent with hypertension and dyslipidemia. Computed tomography (CT) showed a large mass occupying most of the left kidney, consistent with renal malignancy. A left radical nephrectomy with ipsilateral lymph node dissection was performed through left subcostal approach and mobilization of spleen and pancreas. The patient normally recovered after the operation and was discharged four days later.

**Figure 1:**
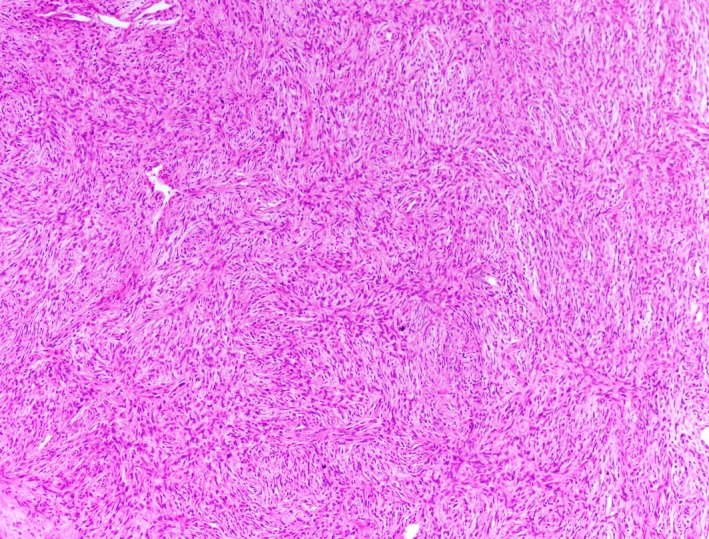
Histopathological features of renal sarcoma demonstrate hypercellular fascicles of spindle cells with elongated, hyperchromatic nuclei, and eosinophilic cytoplasm. Thin, pink, and wavy fibers between the tumor cells are indicative of a sarcoma with fibroblastic differentiation

The pathology indicated malignant sarcoma involving the left kidney and peri‐renal adipose tissue. The mass measured 13.0 × 9.5 × 6.5 cm. There was no involvement of the renal vein or adrenal gland, and the two lymph nodes examined had no evidence of malignancy. Most of the tumor showed hypercellular fascicles of spindle cells with elongated, hyperchromatic nuclei, and eosinophilic cytoplasm. Thin, pink, wavy fibers between the tumor cells were characteristic of collagen and were indicative of a sarcoma with fibroblastic differentiation (Figure 1). Tissue taken adjacent to the main tumor mass showed lobules of mature adipose tissue separated by thick fibrous septa containing scattered atypical cells with enlarged, hyperchromatic nuclei; these latter features are seen in well‐differentiated liposarcoma. Also, the tumor cells were negative for keratin, S‐100, H‐Caldesmon, desmin, CD34, and CD99 by immunohistochemistry.

**Figure 2:**
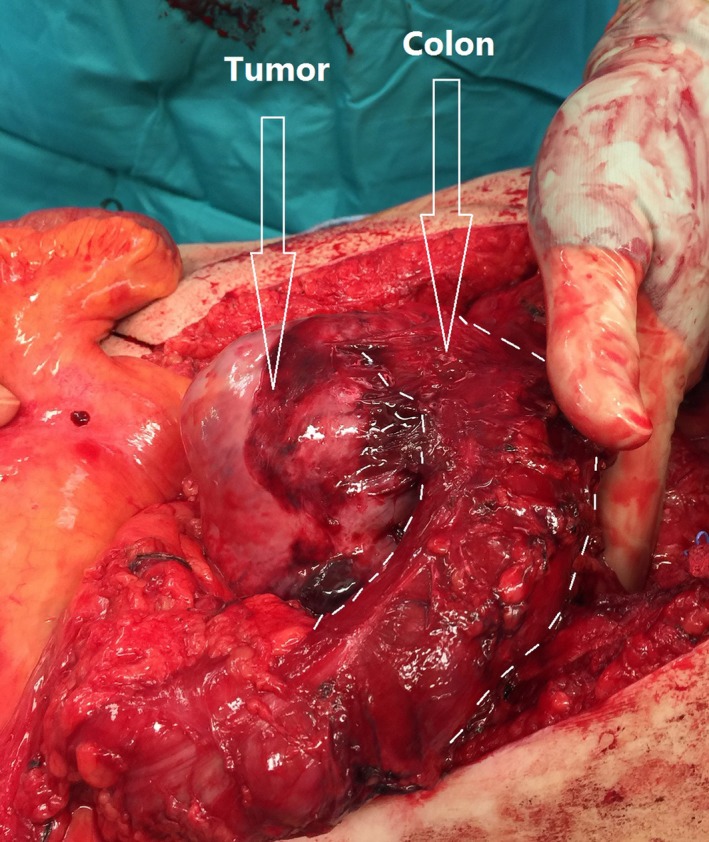
Showing the degree of invasion of the recurring tumor into the colon. The colon was divided at the level of the splenic flexure proximally and at the upper sigmoid distally with primary colo‐colic anastomosis

After the operation, the patient did not receive any adjuvant therapy and remained cancer free for the next two years. After that, he lost follow‐up and presented 11 years later with a CT scan showing a large mass in the left renal fossa adherent to the aorta involving the left diaphragm, psoas muscle, spleen, pancreas, and descending colon. A week after, the patient underwent en bloc excision of the retroperitoneal mass with a segment of the left diaphragm, left psoas muscle, and the descending colon (Figure 2) along with an extensive lymph node dissection and colo‐colic anastomosis. The procedure required meticulous dissection of the mass from the abdominal aorta, spleen, and pancreas. The patient made a speedy recovery and was ambulating in a short time. He was discharged within a week. The latest follow‐up was 18 months after the surgery. The patient enjoyed a good performance status with no evidence of recurrence or metastasis.

**Figure 3:**
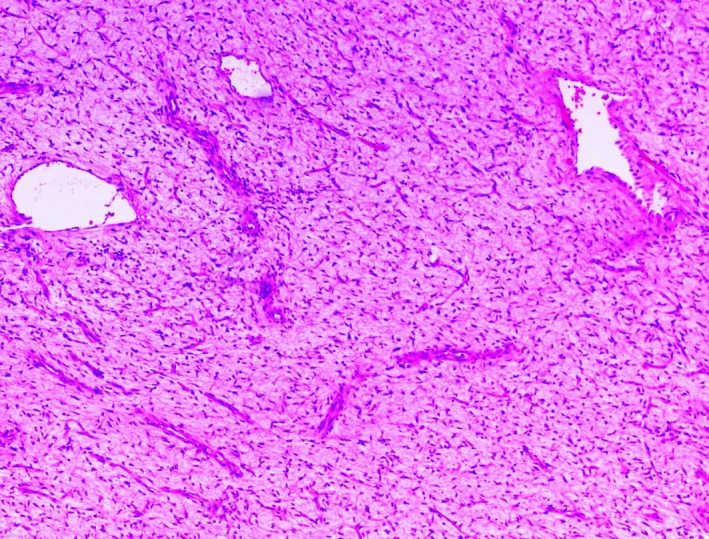
Histopathological characteristics of the recurring tumor depict features of malignant sarcoma with areas of myxoid stroma that contain elongated, thin‐walled blood vessels, resembling the morphology of myxofibrosarcoma

**Figure 4:**
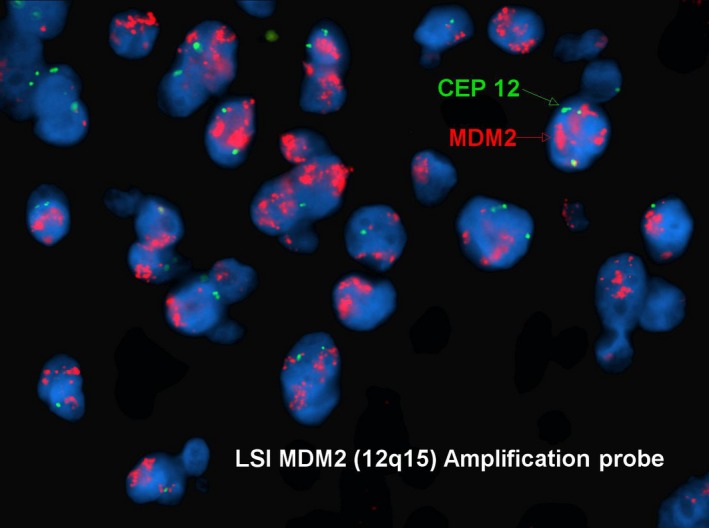
Fluorescent in situ hybridization (FISH) shows amplification of MDM2 gene on the short arm of chromosome 12 (12q15) which strongly supports the diagnosis of dedifferentiated liposarcoma

Pathology of the mass was consistent with a malignant sarcoma (grade 2/3) and portions of the tumor had similar morphological features to the previous case. There were areas with more myxoid stroma that contained elongated, thin‐walled blood vessels, resembling the morphology of myxofibrosarcoma (Figure 3). Fluorescent in situ hybridization (FISH) for MDM2 showed amplification (Figure 4), which strongly supported the diagnosis of dedifferentiated liposarcoma.

Given the similar morphologic features, the original case also likely represents a dedifferentiated liposarcoma. The finding in the initial tumor of an area resembling well‐differentiated liposarcoma also supports the diagnosis. The classic appearance of dedifferentiated liposarcoma is abrupt or gradual transition from well‐differentiated liposarcoma to a non‐lipogenic sarcoma. The non‐lipogenic component can have a broad range of morphologic appearances and frequently mimics myxofibrosarcoma. Finally, all lymph nodes resected (15/15) were negative for malignancy.

## 3 DISCUSSION

Renal sarcoma is a rare malignancy that occurs in only 1% of all kidney cancer.^1^, ^2^ Wang et al analyzed 41 cases of renal sarcoma from 1989 to 2009. The most common histological subtype was leiomyosarcoma in 16 patients (39%), followed by liposarcoma in 12 (29.3%), and other in 13 (31.7%).^3^ Diagnosis of renal sarcoma can be made through radiological and histological techniques by using biomarkers such as desmin, vimentin, neuron‐specific enolase, and S‐100. Radiographic evidence that can be seen on computerized tomography include linear vascularization, aneurismal dilatation, hematoma, or presence of tissue with fat attenuation.^4^ Upon microscopic examination of the mass, areas of compressed cortex, medulla, and renal pelvis can also be found. The areas resembling pleomorphic lipoma may be indistinguishable, but a characteristic spindle cell formation will be observed.^4^

Our patient's tumor overexpressed the MDM2 gene, located on the short arm of chromosome 12. Amplification of MDM2 is the primary feature of well‐differentiated liposarcoma, dedifferentiated liposarcoma, and well‐differentiated osteosarcoma. Amplification of MDM2 can be detected by fluorescence in situ hybridization (FISH) or by an immunohistochemical stain which highlights the nuclei of tumor cells. Abbas et. al. reported 7 cases of spindle cell tumors of the kidney, where a large panel of immunohistochemistry and fluorescence in situ hybridization (FISH) techniques were used to correctly diagnose the pathology of spindle cell sarcoma, using the streptavidin‐biotin‐peroxidase method.^5^ Common biomarkers used were Vimentin, CD99, and BCL‐2 to rule out other sarcomas.^5^ The prognosis of renal sarcoma depends on the size, differentiation, and grade of the tumor. Primary surgical resection with negative margins is the corner‐stone for best outcomes. Extensive surgical resection with wide margins may be the best option for treatment of this rare and aggressive tumor, with clinical follow‐up to monitor for tumor recurrence. With recurrence, wide surgical resection should be attempted if possible to decrease further risk of recurrence.

To our knowledge, our patient is one of the few reported locally advanced renal sarcoma cases who has managed to maintain an excellent performance status for more than 14 years after the initial diagnosis. Mayes et al^4^ reported a case of renal sarcoma that recurred 13 years after radical nephrectomy, but it was inoperable at that time because of the involvement of multiple abdominal organs precluding it from surgical resection.

## 4 CONCLUSION

Renal sarcoma is a rare and aggressive disease. Extensive surgical extirpation with clinical follow‐up post resection is the corner‐stone for best outcomes. Further investigation is needed to define proper treatment guidelines for this disease.

## CONFLICT OF INTEREST

No conflict of interest.

## AUTHOR CONTRIBUTION

The authors listed below have made substantial contributions to the intellectual content of the paper in the various sections described below. MA and GC: involved in conception and design. MA, IZ, and AY: involved in acquisition of data analysis and interpretation of data. MA, IZ, AY, and NM: involved in drafting of the manuscript. GC: involved in critical revision of the manuscript for important intellectual content and involved in supervising the article.
